# The Spectrum of *PRRT2*-Associated Disorders: Update on Clinical Features and Pathophysiology

**DOI:** 10.3389/fneur.2021.629747

**Published:** 2021-03-04

**Authors:** Annamaria Landolfi, Paolo Barone, Roberto Erro

**Affiliations:** Department of Medicine, Surgery and Dentistry “Scuola Medica Salernitana, ” University of Salerno, Baronissi, Italy

**Keywords:** paroxysmal kinesigenic dyskinesia, benign familial infantile seizures, hemiplegic migraine, synaptic dysfunction, cerebellum

## Abstract

Mutations in the *PRRT2* (proline-rich transmembrane protein 2) gene have been identified as the main cause of an expanding spectrum of disorders, including paroxysmal kinesigenic dyskinesia and benign familial infantile epilepsy, which places this gene at the border between epilepsy and movement disorders. The clinical spectrum has largely expanded to include episodic ataxia, hemiplegic migraine, and complex neurodevelopmental disorders in cases with biallelic mutations. Prior to the discovery of *PRRT2* as the causative gene for this spectrum of disorders, the sensitivity of paroxysmal kinesigenic dyskinesia to anticonvulsant drugs regulating ion channel function as well as the co-occurrence of epilepsy in some patients or families fostered the hypothesis this could represent a channelopathy. However, recent evidence implicates PRRT2 in synapse functioning, which disproves the “channel hypothesis” (although PRRT2 modulates ion channels at the presynaptic level), and justifies the classification of these conditions as synaptopathies, an emerging rubric of brain disorders. This review aims to provide an update of the clinical and pathophysiologic features of *PRRT2*-associated disorders.

## Introduction

Paroxysmal kinesigenic dyskinesia (PKD) is a rare disorder with a prevalence estimated at 1:150,000 (1), characterized by recurrent, brief attacks of chorea, dystonia, ballism, or a combination thereof, with preserved consciousness. PKD is the most frequent subtype of paroxysmal dyskinesia (PxD), in which the attacks are by definition triggered by the initiation of voluntary movements, hence *kinesigenic*, but may be further precipitated by non-kinesigenic triggers (2) and usually last <1 min. This disorder manifests during childhood or adolescence, when the frequency of attacks might reach hundreds per day, and usually remits during adulthood (3).

Before the discovery of the genetic basis of PKD, it was emphasized that it shared some clinical features with other episodic neurological disorders, including periodic paralyses and epilepsy, often caused by mutations in ion channels. In fact, it was observed that patients with PKD have a dramatic response to antiepileptic drugs (AED) and that epilepsy may co-occur in the same individual or in the family. Such empirical data suggested a common pathophysiological basis for these disorders, leading to a belief that PKD could represent a channelopathy (4, 5).

However, the identification of proline-rich transmembrane protein 2 (*PRRT2*; OMIM^*^614386) as the main causative gene for PKD (6) and related disorders (7) argued against the “ion channel hypothesis” (8). In fact, subsequent research has implicated PRRT2 in synapse functioning, which justifies the classification of *PRRT2*-associated conditions as “synaptopathies,” an emerging rubric of brain disorders (9). Moreover, *PRRT2* mutation screening in other episodic neurological disorders has largely expanded its clinical spectrum.

We here aim to provide an update on the clinical and pathophysiologic features of *PRRT2-*associated disorders.

## Clinical Aspects

### Paroxysmal Dyskinesia

*PRRT2* mutations account for most cases of PKD (10). The phenotype features attacks of short duration (<1 min), consisting of choreic, dystonic, and/or ballistic movements, which are triggered by sudden movements, intention to move, and/or acceleration, hence kinesigenic. Interictal neurological examination is unrevealing in most cases (3).

Although virtually all patients report a kinesigenic trigger, about 40% of them might have additional non-kinesigenic triggers including anxiety, startle, sleep deprivation, and, seldom, sustained exercise (10). This demonstrates that there is clinical overlap with other PxD subtypes [i.e., paroxysmal non-kinesigenic dyskinesia (PNKD) and paroxysmal exercise-induced dyskinesia (PED)], reinforcing the suggestion that the clinical description of PxD should not be solely tailored on the type of trigger, but should include duration of the attacks and response to AED, especially carbamazepine (CBZ). In fact, at variance with classic PNKD and PED (11, 12), *PRRT2-*PxD are brief in duration and show an exquisite response to CBZ (2, 13). The latter feature as well as younger age at onset (around 9 years of age) and familial clustering of PKD, epilepsy and/or other rarer phenotypes (see below) are predictive of *PRRT2* mutations (3, 14, 15). A single study reported this might be true for choreic phenomenology and bilateral distribution of the attacks (15) whereas presence of preceding sensory aura is not indicative of *PRRT2* mutations (10, 15).

Interestingly, *PRRT2* mutations have also been described in cases with isolated paroxysmal hypnogenic dyskinesia (PHD) (16), a fourth PxD form in which attacks occur during sleep without identifiable triggers, which has been increasingly discarded as a PxD subtype following the evidence that autosomal dominant frontal lobe epilepsy (ADFLE) was the underlying etiology in most cases (17). The importance of this observation is 2-fold: (1) it brought the re-inclusion of PHD as an additional PxD subtype, beyond PKD, PNKD, and PED(3); and (2) it demonstrated that additional clinical features beyond the trigger of the attacks (i.e., duration of the attacks and responsivity to CBZ) might be predictive of the underlying genetic deficits.

Although PKD attacks might be violent (18), the condition is considered relatively benign as there is a tendency for remission during adulthood. CBZ is the first line option (50–600 mg) but, according to patient profile and occurrence of side effects (19, 20), other AED including zonisamide (21), topiramate (22), lamotrigine (23), and levetiracetam (24) can be considered.

### Epileptic Disorders

*PRRT2* mutation have also been associated to self-limited familial infantile epilepsy, most commonly referred to as benign familial infantile seizures (BFIS) (25). It is an autosomal-dominant epileptic disorder in which non-febrile convulsions start in the first 12 months of life, have a good response to AED, and have a favorable prognosis with remission before age two (1). Seizure phenomenology consists of focal motor seizures starting with gaze staring, motor arrest and head deviation, hypertonia and cyanosis, which usually occur in clusters and might have secondary generalization (25–27). The ictal EEG often shows parieto-occipital epileptic activity that may eventually generalize (28). Bilateral tonic-clonic or absence seizures and benign myoclonus of infancy have been rarely described (27, 29, 30). Conversely, *PRRT2* mutations are not found in families with atypical infantile seizures such as later seizure onset or offset, more severe seizures, or multiple seizure type (29).

**Figure 1:**
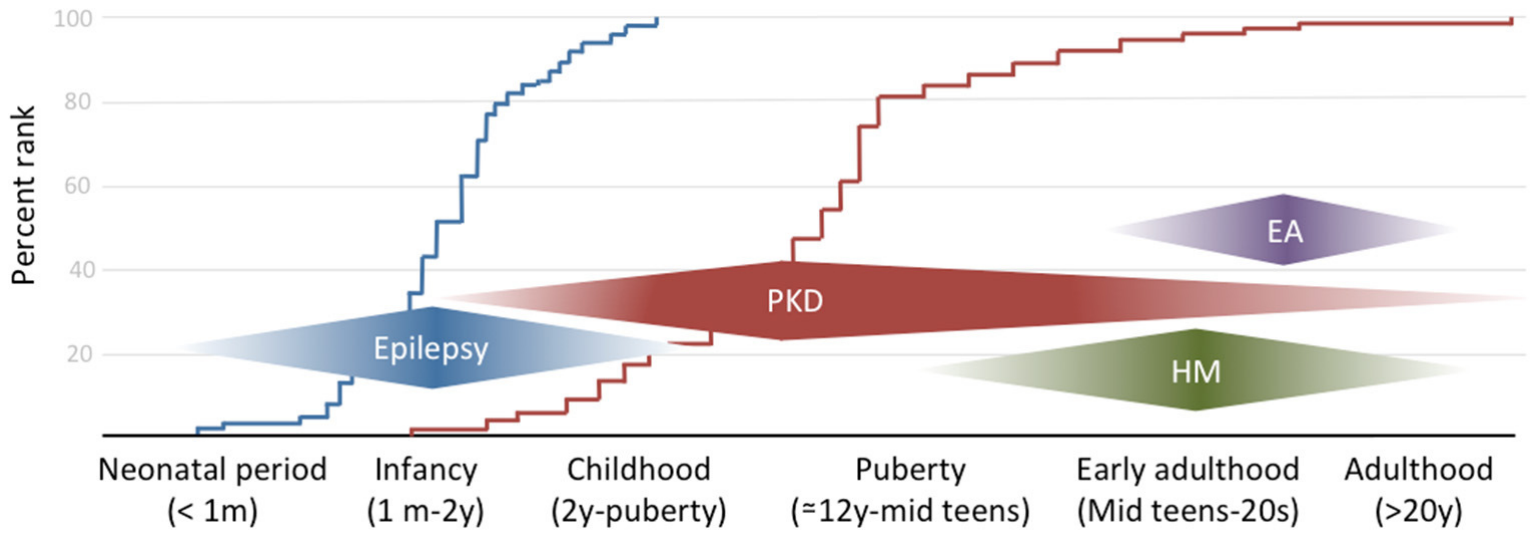
The clinical spectrum of *PRRT2*-associated disorders by onset age. The curves on the background reflect the empirical distribution of age at onset for the two commonest phenotypes (i.e., epilepsy in blue and paroxysmal dyskinesia in red) based on references (10, 26, 27).

The combination of infantile convulsion and paroxysmal kinesigenic dyskinesia in the same subject configures the paroxysmal kinesigenic dyskinesia with infantile convulsions (PKD/IC) syndrome, formerly known as the infantile convulsions with choreoathetosis (ICCA) syndrome. This syndrome combines an epileptic disorder presenting in the first year of life and usually remitting within 2 years of age, with the appearance later in life of PKD (7) (Figure 1).

### Other Clinical Syndromes

Beyond the clinical syndromes described above, heterozygous *PRRT2* mutations have been associated with hemiplegic migraine (HM), with episodic ataxia (EA), and, anecdotally, with a variety of paroxysmal movement disorders, not strictly fulfilling the criteria for PxD. In most cases, such phenotypes occur in combination with the classic syndromes associated with *PRRT2* mutations (i.e., epilepsy and PxD), hence the importance of their recognition to aid the differential diagnosis, but can seldom occur in isolation (28).

HM is a rare subtype of migraine with aura, in which attacks are associated with transient motor weakness or hemiparesis. Age at onset might be in late childhood but most commonly is during puberty or early adulthood (Figure 1), with attacks frequency ranging from a few per week to 1 per month. Attacks might be triggered by stress, anxiety, light, or heat (31, 32). Motor aura duration can be as long as 72 h, coherently to what previously reported about familial and sporadic HM (33). Interestingly, in one case migraine attack duration was reported to be as short as 15 min (32). To our knowledge, this disproportion between aura and migraine duration has never been described in other forms of HM and might arguably constitute a red flag for *PRRT2* mutations along with CBZ sensitivity, which has been described also with this phenotype (34). Despite HM being the commonest reported phenotype, *PRRT2* mutations can also manifest with other, non-complicated, types of migraine, with or without aura (35), generally associated with epilepsy and/or PKD. However, given the high prevalence of migraine in the general population, a specific association with *PRRT2* mutations deserves further studies.

Very rarely, heterozygous *PRRT2* mutations have been reported to cause EA (31). Given the rarity of this presentation, which tends to occur later than the other *PRRT2*-associated syndromes, genotype-phenotype correlations have not been elucidated nor is it possible to identify any red flags to suspect *PRRT2* mutations in patients with EA. Nonetheless, it should be noted that extensive *PRRT2* screening in patients with EA has not been performed so that the exact figure of *PRRT2* carriers compared to other genes causing EA is unknown.

Anecdotally, *PRRT2* mutations have been described with other paroxysmal movement disorders, including paroxysmal torticollis of infancy (co-occurring with epilepsy in a patient with positive family history for PKD and HM) (36), intermittent non-epileptic head drops (again co-occurring with epilepsy) (30), and the “galloping tongue” syndrome (i.e., involuntary tongue movements only appearing upon tongue protrusion, which can be arguably deemed as a *forme fruste* of PKD) in a *PRRT2* mutation carrier with a positive family history for PxD (37).

Beyond paroxysmal neurological disorders, there is preliminary evidence that heterozygous *PRRT2* mutations might also cause intellectual disability and/or developmental delay (26, 27, 38), a suggestion supported by their invariable presence in cases with 16p11.2 deletions (39–41) and homozygous *PRRT2* mutations (27, 42–44). The latter evidence, along with initial demonstrations that *PRRT2* mutations can possibly cause brain structural alterations (27, 44), is of crucial importance since it implicates PRRT2 in neurogenesis and brain development, as discussed below. However, it should be noted that, in the context of 16p11.2 deletions, phenotype severity might be owing to deletion of adjacent genes and, therefore, the association of intellectual disability and heterozygous *PRRT2* mutations (26, 27, 38) requires additional confirmation.

## Pathophysiologic Aspects

*PRRT2* is located on chromosome 16p11.2 and consists of four exons, three of which encode a protein of 340 amino acids, the proline-rich transmembrane protein 2, that is composed of a proline-rich, extracellular N-terminal domain and a membrane-bound C-terminal domain.

Different mutation types (mainly nonsense, missense, or deletion), the frameshift mutation NM_145239 c.649dupC (p.Arg217Profs^*^8) that leads to a premature stop codon being the most common (reported in more than 75% of carriers, which suggests a mutational hotspot), are thought to be responsible for a protein loss-of-function mechanism causing the disorder due to gene haploinsufficiency (28). This would be supported by the evidence that the c.649dupC-derived mRNA is degraded by nonsense-mediated decay, not being therefore translated into a protein (28). In most instances, *PRRT2* mutations are inherited in an autosomal dominant fashion and account for familial cases, but *de novo* mutations have been also described and are estimated to occur in about 5% of cases.

**Figure 2:**
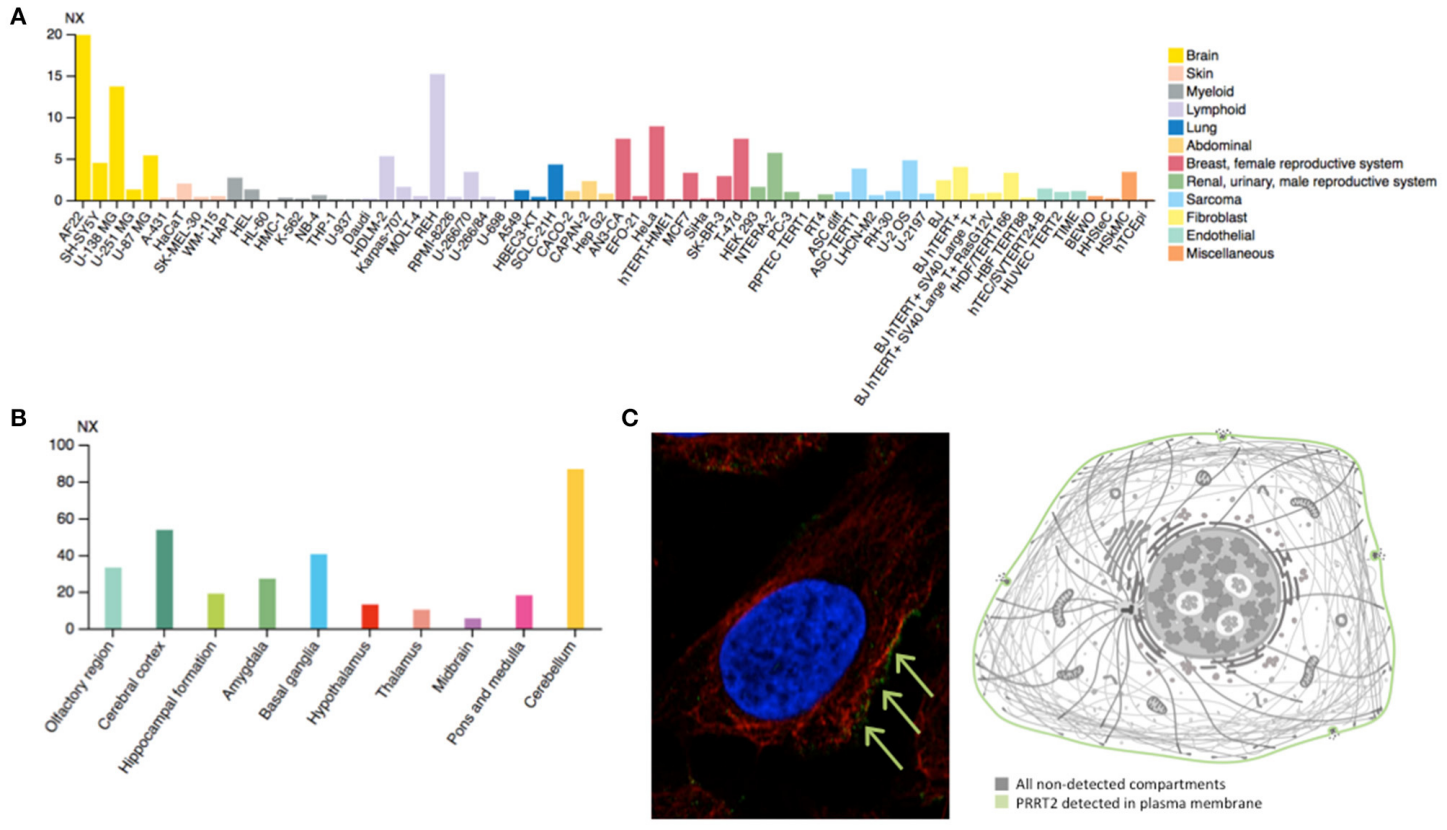
**(A)** Estimated transcript abundance in different cell lines, ordered by organ of phenotypic resemblance, showing that PRRT2 is mostly expressed in brain cell lines. **(B)** PRRT2 RNA expression levels in 10 brain areas obtained by combining data from two transcriptomics dataset (GTEx and FANTOM5), showing that PRRT2 is highly expressed in the cerebellum, cerebral cortex, and basal ganglia. **(C)** Subcellular location of PRRT2 obtained in AF22 cell line by indirect immunofluorescence microscopy (i.e., an antibody-based protein-visualization technique, in which nucleus is stained in blue, microtubules in red and the protein of interest in green) showing that PRRT2 localizes to the plasma membrane, as depicted in the cartoon. NX = consensus normalized expression. For details see https://www.proteinatlas.org/.

PRRT2 is highly expressed in the human brain (Figure 2A), especially in the cerebral cortex, basal ganglia, and cerebellum (Figure 2B), which matches with the expected locations based on the aforementioned phenotypes. This mirrors the evidence stemming from animal model studies in mice, demonstrating highest PRRT2 mRNA levels in the same brain areas (45). However, mice basal ganglia and neocortex are found to exhibit relatively low mRNA and high protein levels. Taking into account the predominant presynaptic expression of PRRT2 (see below), this would indicate that neocortex and basal ganglia might be mostly targets of projections arising from other areas rich in PRRT2-expressing neurons (i.e., the cerebellum) (46).

At the cellular level, PRRT2 localizes at the plasma membrane (Figure 2C), especially at the presynaptic level, and is mainly expressed in glutamatergic neurons (28). This protein was first described to interact with 25 kDa Synaptosomal-Associated Protein (SNAP25), which is involved in Ca^2+^-mediated neurotransmitter release through its function as a t-SNARE protein, its role in synaptic endocytosis that impacts the availability of synaptic vesicles at the readily releasable pool, and its regulation of voltage-gated ion channels (28). Subsequent studies have further showed that PRRT2 interacts with other synaptic proteins, such as the Vesicle Associated Membrane Protein 2 (VAMP2) and the synaptotagmins Syt1 and 2 (47), which again implicates PRRT2 in the Ca^2+^-sensing machinery involved in the final steps of neurotransmitter release. Moreover, *PRRT2*-silenced neurons exhibited a strong impairment in synchronous, evoked neurotransmitter release in excitatory synapses, with no effect on asynchronous release, which results in marked increase of the asynchronous/synchronous release ratio and suggests that the vescicle fusion mechanism is not altered *per se* and that a specific defect in coupling Ca^2+^ influx to exocytosis is the most likely pathomechanism (47, 48). Moreover, short-term potentiation (STP) phenomena in response to stimulation trains of short duration (i.e., 2 s) and of increasing frequency (i.e., 5–40 Hz) that promote Ca^2+^ build-up in the nerve terminals showed opposite effects on excitatory (i.e., glutamatergic) and inhibitory (i.e., GABAergic) synapses. Indeed, an increased facilitation in excitatory transmission and an increased depression in inhibitory transmission was observed (49), suggesting an excitation/inhibition imbalance in the STP frequency domain underlying a state of hyperexcitability/instability in neuronal networks expressing the mutant protein. Such network dynamics, which are highly dependent on the STP of excitatory and inhibitory synapses rather than on their basal transmission properties, might directly explain the paroxysmal nature of the dyskinesia that would manifest only when the network is challenged by a kinesigenic trigger.

Although PRRT2 is intimately implicated in the Ca^2+^-sensing machinery regulating neurotransmission at the synaptic level, it has additional physiologic roles that likely explain some clinical features observed in humans. In fact, PRRT2 has been demonstrated to negatively regulate voltage-gated Nav1.2 and Nav1.6 channels by modulating their voltage-dependent state of inactivation and their recovery from inactivation (50). Studies conducted on induced pluripotent stem cell-derived neurons from homozygous patients and on primary neurons obtained from *PRRT2* knockout mice, showed an increase of Na^+^ currents resulting in markedly augmented spontaneous firing, which was even higher when neurons were challenged with supra-threshold, high-frequency stimulation (50). In both conditions, abnormal firing was fully reverted by the reintroduction of wild-type PRRT2 (50). The latter evidence has two important implications: (1) beyond the aforementioned synaptic dysfunction, the disturbance in cellular excitability by lack of negative modulation of Na^+^ channels might further explain the predominant paroxysmal character of *PRRT2*-associated disorders and the dramatic effectiveness of sodium channel modulators, such as CBZ (51); and (2) it would represent a mechanistic crossover between synapthopathies and channelopathies underlying paroxysmal neurological disorders (5), since mutations in *SCN8A* encoding for the voltage-gated, Na^+^ channel, alpha subunit Nav1.6, can also cause the association of PxD with epilepsy (52).

Finally, several layers of evidence implicate *PRRT2* in brain development. Studies conducted on developing mouse brain and primary neuronal cultures demonstrated that PRRT2 is highly expressed during the early stages of development (47), in which intense synaptogenesis occurs. Using these models, it was demonstrated that *PRRT2* silencing negatively affected synaptic connections, which can be interpreted as a developmental effect (47). In mouse embryos, *in utero PRRT2* knocking out in cortical neurons causes a delay in neuronal migration and defects in synaptic development (53). Furthermore, examination of PRRT2 expression pattern in the developing murine nervous system revealed a relative decline during adulthood (51). These results, along with the evidence that homozygous mutations can cause in humans developmental delay, intellectual disability, and brain structural alterations, strongly support an additional role of *PRRT2* in neurodevelopment. Indeed, one recent study has showed that in primary hippocampal neurons, *PRRT2* silencing affects synaptic actin dynamics, leading to defects in dendritic spine density and maturation, through the defective interaction with cofilin, an actin-binding protein that is abundantly expressed at the synaptic level (54). Interestingly, the expression of a cofilin phospho-mimetic mutant was able to rescue *PRRT2*-dependent defects in synapse density, spine number, and morphology, but not the alterations observed in neurotransmitter release (54), which confirms an independent mechanism arguably underpinning the neurodevelopmental phenotype. The observed plateauing of PRRT2 expression during adulthood further supports the existence of critical age windows for the occurrence of related phenotypes (Figure 1) and their self-limiting character (5, 28).

## Conclusions

In recent years, significant progress has been made leading to a precise characterization of the clinical spectrum of *PRRT2-*associated disorders as well as increased understanding of the underlying pathophysiology. Thus, *PRRT2* mutations share synaptic dysfunction as the main pathomechanism with other “synaptopathies,” causative of a number of neurological, psychiatric, and childhood developmental disorders (55).

Nonetheless, there remain critical open questions for future investigation. For instance, genotype-phenotype correlations in humans are not strict, as suggested by the incomplete penetrance, which has been estimated to be about 75–90% for epilepsy and only 50–61% for PKD in pediatric cohorts (26, 56). Data about adult patients with PKD are lacking. However, it should be noted that penetrance figures might differ in different populations of children and adults cohorts given the age-dependent natural history of *PRRT2*-disorders. In fact, whereas the pattern of PRRT2 brain expression (Figure 2B) matches with the human phenotypes, it should be noted that the figures derive from the analysis of a set of adult-derived brain regions, thus preventing the recognition of potentially critical temporal windows. The observed dissociation in terms of age at onset between the epilepsy and PKD phenotypes might in fact suggest an expression pattern shift across different brain regions during development (57). Moreover, the clinical variability of the same mutation, which can cause both epilepsy and movement disorders either in a given patient or family, or in separate families, is another aspect that should be investigated by exploring the putative role of modifiers, genetic or otherwise.

Although the brain area where *PRRT2* is mostly enriched is the cerebellum, ataxia represents the least common phenotype in humans, raising the question of whether or not dysfunction of cerebellar activity is involved also in the genesis of PxD and epilepsy. Evidence from animal *PRRT2* models suggest that local cerebellar hyperexcitability would be sufficient to generate involuntary dyskinesia (58) resembling the PKD phenotype in humans, learning difficulties (59) as observed with patients carrying biallelic *PPRT2* mutations, and, to some extent, also an epileptic susceptibility (46). These results might support the hypothesis that cerebellar deficits might in fact drive both epilepsy (60) and movement disorders, especially dystonia (61), but additional research is needed to confirm whether this applies to *PPRT2* mutations in humans.

Finally, although a loss-of-function mechanism seems most likely, at least for the commonest *PRRT2* mutations including the frameshift c.649dupC (58), it is still possible that other less frequent mutations that are unlikely to undergo nonsense-mediated decay could act through a dominant-negative mechanism, perhaps by the interaction with the protein encoded by the unaffected allele, and this needs to be specifically investigated.

Notwithstanding, a great advance into the understanding of the mechanisms underpinning *PRRT2*-associated disorders has been recently made. This will hopefully drive an effort to move from symptomatic treatments to therapeutic options targeting their specific pathophysiologic alterations and might further facilitate research in other paroxysmal neurological disorders associated with synaptic dysfunction.

## Author Contributions

RE and PB: conceptualization and supervision. RE: methodology. RE and AL: resources, writing - original draft, and writing - review & editing. All authors contributed to the article and approved the submitted version.

## Conflict of Interest

The authors declare that the research was conducted in the absence of any commercial or financial relationships that could be construed as a potential conflict of interest. The Handling Editor declared a past co-authorship with one of the authors RE.
